# Supplementary material for: A combination of transcription factors mediates inducible interchromosomal contacts
Source: eLife. 2019 May 13;8:e42499. doi: 10.7554/eLife.42499 (PMC6548505; doi:10.7554/eLife.42499)
Supplement: Supplementary file 3. [file elife-42499-supp3.docx]

**96-well yeast transformation protocol**

**Day 1**1. Array strains into shallow 96-well plate, resuspending one colony of each strain in 100 ul YPD.
2. Incubate overnight at 30C.
 **Day 2**3. Transfer 45 ul into each of two deep 96-well plate with 400 ul YPD per well. One plate will be used to make glycerol stocks.
4. Incubate overnight at 30C.
 **Day 3**5. With one deep 96-well plate, make glycerol stocks by resuspending each well by pipetting and then transferring 80 ul to a U-bottom 96-well plate with 40 ul of 50% glycerol already added. Store at -80C.
6. With the other deep 96-well plate, carefully remove 360 ul (180 ul twice) supernatant by pipetting.
7. Add 200 ul 2xYPAD and resuspend by pipetting.
8. Incubate at 30C for 1.5 hrs.
9. During this incubation, denature salmon sperm DNA (ssDNA) by placing in 98C heat block for 5 min, and then place on ice.
10. Pellet by centrifuging at 2000g for 2 min.
11. Remove 200 ul supernatant by pipetting.
12. Add 180 ul water and resuspend by pipetting.
13. Pellet by centrifuging at 2000g for 2 min.
14. Remove supernatant by pipetting.
15. Add 180 ul 0.1M LiAc and resuspend by pipetting.
16. Pellet by centrifuging at 2000g for 2 min.
17. Remove supernatant by pipetting
18. Add 120 ul 50% PEG-3350 + 18 ul 1.0M LiAc + 5 ul denatured ssDNA (pre-mixed together: 26 ml 50% PEG-3350, 3.9 ml 1.0M LiAc, 1.083 ml ssDNA; use 143 ul per well)
19. Resuspend by pipetting.
20. Add 20 ul DNA, mix by pipetting up and down.
21. Resuspend by gently vortexing.
22. Incubate at 30C for 30 min.
23. Resuspend by gently vortexing.
24. Incubate at 42C for 40 min in water bath.
25. Pellet by centrifuging at 2000g for 2 min.
26. Remove 180 ul supernatant by pipetting.
27. Add 160 ul water, resuspend by pipetting
28. Pellet by centrifuging at 2000g for 2 min
29. Remove supernatant by pipetting
30. Add 100 ul water, and resuspend by pipetting.
31. Add 1 ml C-ura+2% glucose. Pipette up and down to mix.
32. Incubate at 30C overnight.
 **Day 4**33. Remove supernatant by pipetting.
34. Add 50 ul water, resuspend by pipetting/shaking, transfer to shallow 96-well plate
35. Pin/pipette onto C-ura+2% glucose plate(s)
36. Incubate at 30C for 2-4 days
37. Pick colonies for PCR verification and grow in shallow 96-well plate with 150 ul C-ura+2% glucose
